# Exploitation of the Host Cell Membrane Fusion Machinery by *Leishmania* Is Part of the Infection Process

**DOI:** 10.1371/journal.ppat.1005962

**Published:** 2016-12-08

**Authors:** Christine Matte, Albert Descoteaux

**Affiliations:** INRS-Institut Armand-Frappier and Centre for host-parasite interactions, Laval, Quebec, Canada; University of Wisconsin Medical School, UNITED STATES

Successful vacuolar pathogens have developed sophisticated strategies to hijack the endomembrane system of host cells, sabotage their signal transduction pathways, and evade antimicrobial responses. The protozoan parasite *Leishmania*, the causative agent of leishmaniases in humans, is particularly adept at transforming the macrophage into a hospitable host cell. Here, we describe how *Leishmania* promastigotes subvert the macrophage membrane fusion machinery to create an intracellular compartment favorable to the establishment of infection and to manipulate host immune responses.

## What Is the Membrane Fusion Machinery?

Cellular functions such as phagocytosis and cytokine secretion rely heavily on a complex network of vesicle trafficking pathways that interconnect most membrane-bound intracellular compartments [1]. A critical step in the exchange of cargoes between vesicles in this network is the process of membrane fusion, which is mediated by SNARE (soluble *N*-ethylmaleimide-sensitive factor attachment protein receptor) proteins. This superfamily of integral and peripheral membrane proteins displays the distinctive SNARE motif, a stretch of heptad repeats that form a coiled-coil structure with a conserved arginine (R) or glutamine (Q) residue at the central “0” layer. Fusogenic SNARE complex formation requires the parallel association of an R-SNARE domain on the vesicle membrane with three cognate Q-SNARE domains on the target compartment. The subsequent “zippering” of this four-helix bundle from the membrane-distal amino termini towards the membrane-proximal carboxyl termini brings the two apposed membranes into close proximity and provides sufficient mechanical force to overcome the energy barrier for the formation of the fusion pore [1].

Whereas SNAREs make up the core of membrane fusion machinery, a large number of additional proteins are required for the spatiotemporal orchestration of the entire process [2]. For instance, the family of Rab GTPases are master regulators of virtually all events leading to membrane fusion. Indeed, Rab effectors include motor proteins (for vesicle trafficking along the cytoskeleton) and membrane tethers (for the initial, loose attachment to the target compartment). A set of chaperones, such as the proteins of the Sec1/Munc18 (SM) family, Munc13 and Complexins, oversee the timing of cognate SNARE pairing by restricting SNARE accessibility at first, then initiating partial SNARE complex formation and keeping it in a “release-ready” state until the appropriate moment for membrane fusion. The final trigger for pore formation is provided by synaptotagmins (Syts), a family of calcium-sensing membrane proteins that control fusion via interactions with SNARE proteins and membrane lipids. Ultimately, SNARE complexes are disassembled by the ATPase NSF (*N*-ethylmaleimide-sensitive factor) and the adaptor protein α-SNAP (soluble NSF attachment protein).

## What Is the Role of Vesicle Trafficking and Membrane Fusion during the Phagocytic Process?

Binding and internalization of infective *Leishmania* stages to macrophages involves multiple phagocytic receptors [3]. Phagocytosis of those large particles requires the expense of a considerable amount of plasma membrane for pseudopod extension around the prey. Various membrane-bound intracellular compartments lend a hand to this process by fusing with the cell surface and rapidly providing endomembrane required for particle engulfment [4]. Focal exocytosis of recycling endosomes, for instance, contributes not only to phagocytosis but also allows for rapid secretion of preformed inflammatory cytokines including TNF and IL-6 [5]. Fusion of recycling endosomes with the cell surface is mediated by the R-SNARE VAMP3 (vesicle-associated membrane protein 3) [1, 4] and is regulated by Syt V [6]. Interestingly, Syt XI also localizes to recycling endosomes and is recruited to nascent phagosomes, but acts as a negative regulator of phagocytosis and cytokine secretion [7]. Late endosomes and lysosomes assist large particle phagocytosis as well, in a VAMP7- and Syt VII-dependent manner [1, 4, 8]. Contribution of the endoplasmic reticulum (ER) as a source of endomembrane varies according to the nature of the phagocytosed particle and requires the ER Q-SNARE Stx18 [4, 9].

Upon the completion of particle internalization, the phagosome undergoes a series of “kiss-and-run” fusion and fission events with vesicles of the endocytic pathway, culminating in the creation of a highly microbicidal and immunologically competent compartment, the phagolysosome. Various components of the membrane fusion machinery participate in the genesis of this organelle, including the endosomal R-SNARE VAMP8 for the recruitment of the NADPH oxidase NOX2 [10], Syt V for the acquisition of the v-ATPase (vesicular proton-ATPase) [11], and the ER R-SNARE Sec22b for the recruitment of ER components required for antigen crosspresentation [12].

## Do *Leishmania* Parasites Disrupt the Membrane Fusion Machinery to Tamper with Macrophage Responses?

**Fig 1:**
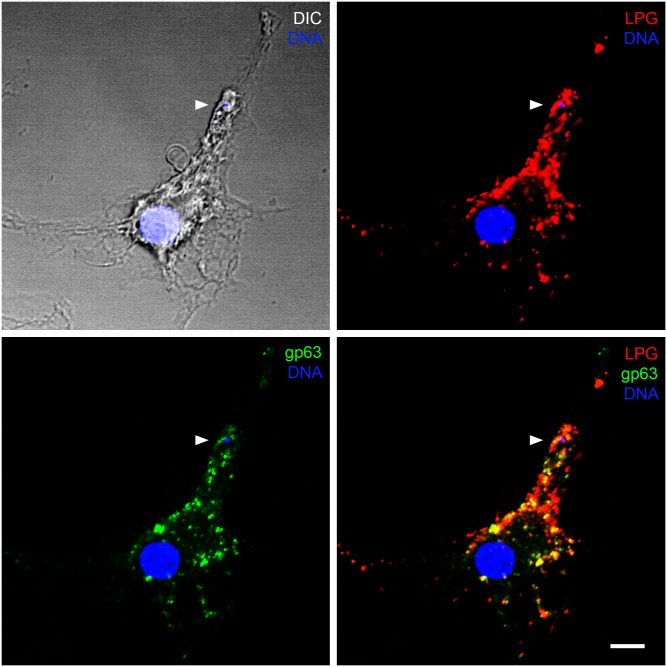
Distribution of *L*. *major* virulence factors LPG and GP63 across an infected macrophage. Murine primary macrophages were infected for 6 h with *L*. *major* promastigotes, fixed and stained for confocal immunofluorescence microscopy using DAPI (DNA, blue) and antibodies against LPG (red) and GP63 (green). Arrowheads point to parasite nuclei. Scale bar = 5 μm.

Pathogens use a variety of tactics to manipulate membrane fusion and vesicle trafficking to cause disease [13]. The intracellular bacteria *Chlamydia* and *Legionella*, for instance, produce proteins with SNARE-like motifs that interact with host SNAREs and inhibit SNARE-mediated membrane fusion. The best-known example is the specific cleavage of SNAREs by clostridial neurotoxins, which are potent blockers of neurotransmission in peripheral cholinergic nervous system synapses [14]. *Leishmania* promastigotes use two abundant surface GPI-anchored virulence factors to interfere with vesicle trafficking and fusion: GP63 (glycoprotein 63), a zinc-dependent metalloprotease, and LPG (lipophosphoglycan), a polymer of repeating Gal_β1,4_Man_α1_-PO_4_ units. Upon internalization of the parasites, GP63 and LPG are rapidly redistributed throughout infected cells (Fig 1). Akin to the clostridial neurotoxins, GP63 cleaves components of the host cell membrane fusion machinery, including VAMP3, VAMP8, and Syt XI (Table 1) [10, 15]. The consequences of these cleavage events are diverse. In macrophages and dendritic cells, processing of exogenous antigens for crosspresentation on MHC I molecules is controlled by the NADPH oxidase NOX2: phagosome oxidation prevents excessive acidification and destruction of peptides destined for recognition by T cells [16]. Since VAMP8 is involved in the recruitment of NOX2 to phagosomes, GP63-mediated cleavage of VAMP8 results in increased phagosomal proteolytic activity, ensuing in defective crosspresentation of *Leishmania* antigens to T cells [10]. In parallel, during a noncanonical autophagic process referred to as LC3-associated phagocytosis (LAP), NOX2-mediated phagosomal oxidation promotes the recruitment of the autophagy-related protein LC3 to a subset of phagosomes. Several roles have been attributed to LAP, including increased phagosomal microbicidal activity and enhanced antigen presentation on MHC II molecules [17]. By cleaving VAMP8 and preventing phagosomal recruitment of NOX2, GP63 allows *Leishmania major* promastigotes to evade LAP [18], possibly contributing to the impairment of phagosome maturation and further inhibiting antigen presentation to T cells. Consistent with the role of Syt XI as a negative regulator of cytokine secretion, cleavage of this endosomal protein by GP63 from *L*. *major* promastigotes increases the postinfection release of TNF and IL-6 [15]. These proinflammatory cytokines are responsible for the augmentation of neutrophil and inflammatory monocyte influx to the parasite inoculation site, which contributes to the spread and maintenance of infection.

**Table 1:** Components of the host cell membrane fusion machinery targeted by *Leishmania* parasites. Subcellular localization and function in macrophages of each host protein are indicated.

Targeted host protein	Subcellular localization	Functions	Modification	Parasite factor involved	Ref.
VAMP3	Recycling endosomes	Focal exocytosis of recycling endosomes	Proteolyic cleavage	GP63	[10]
		Cytokine secretion			
VAMP8	Late endosomes and lysosomes	Recruitment of NOX2	Proteolyic cleavage	GP63	[10, 18]
		Crosspresentation			
		LC3-associated phagocytosis			
Syt V	Recycling endosomes	Focal exocytosis of recycling endosomes	Exclusion from the phagosome	LPG	[11]
		Recruitment of vATPase			
Syt XI	Recycling endosomes	Negative regulator of cytokine secretion and phagocytosis	Proteolyic cleavage	GP63	[15]

Insertion of LPG into host cell lipid microdomains causes remodeling of the phagosome and delays its maturation into a highly microbicidal phagolysosome, as a result of reduced fusogenicity towards late endosomes and lysosomes [19]. The current model of LPG-mediated phagosome remodeling is that LPG disrupts membrane lipid microdomains and thereby interferes with the clustering of host molecules at these sites [19]. For instance, SNAREs and other members of the membrane fusion machinery are typically concentrated in cholesterol-rich membrane rafts [1]. Therefore, one consequence of LPG-mediated microdomain disorganization is the exclusion of Syt V from the phagosome (Table 1). This, in turn, abrogates v-ATPase recruitment and impedes phagosome acidification [11].

Aside from GP63-mediated cleavage and LPG-induced phagosomal exclusion of SNAREs and Syts, *Leishmania* parasites also target components of the membrane fusion machinery at the transcriptional level. Infection of human macrophages with *L*. *major* or *L*. *amazonensis* leads to the up-regulation of Syt II, VI, and VIII [20]. The underlying mechanism, including the parasite factors and/or host proteins involved, and the repercussions on PV (parasitophorous vacuole) formation and infection outcome have yet to be uncovered. Of note, the genome of *L*. *major* encodes a repertoire of 27 putative SNARE proteins, which can be segregated into R-SNAREs and Q-SNAREs based on the classification system used for other organisms. Most of these proteins display the expected structural characteristics of functional SNAREs, while a few show peculiarities. For instance, four proteins lack a predicted transmembrane domain or GPI-anchor, and only two out of the four possess potential lipidation sites for attachment to membranes [21]. Whether any of these 27 putative SNAREs play a role in the manipulation of host cell fusion events by *Leishmania* parasites is unknown.

## Do *Leishmania* Parasites Promote Vesicle Fusion to Drive PV Formation?

**Fig 2:**
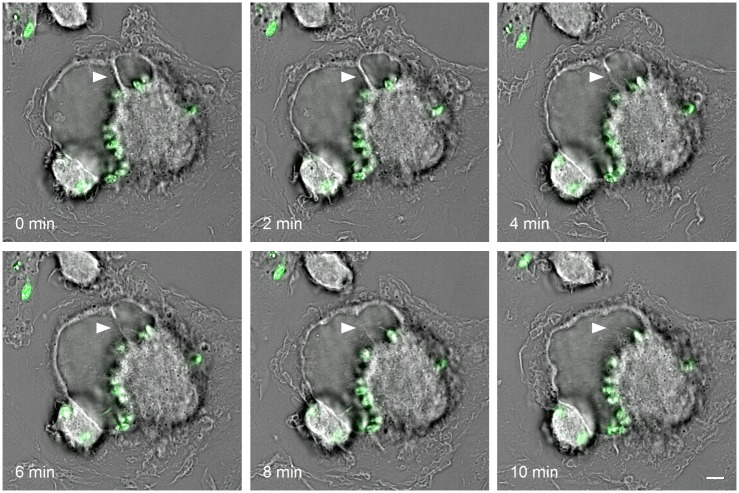
Two communal vacuoles undergoing homotypic fusion. Murine primary macrophages were infected for three days with carboxyfluorescein succinimidyl ester (CFSE)-stained *L*. *amazonensis* parasites (green) and then monitored every two minutes by live-cell imaging. Arrowheads point to the septum between two parasitophorous vacuoles throughout the fusion process. Scale bar = 5 μm.

Old World *Leishmania* species (*L*. *major*, *L*. *donovani*, and *L*. *tropica*) reside in small, tight-fitting PVs that undergo fission shortly after parasite replication, therefore rarely containing more than a single amastigote. On the other hand, the establishment of a successful infection by New World species (*L*. *mexicana*, *L*. *amazonensis*, and *L*. *pifanoi*) requires the formation of spacious, communal vacuoles that can harbour numerous parasites. Our understanding of the mechanisms allowing the development of individual versus communal PVs is very limited. Both types of PVs continuously interact with the host cell reservoir of acidic [22] and ER-derived vesicles [23], most likely to accommodate for the high membrane demand. Biogenesis of large communal PVs involves homotypic fusion between smaller PVs (Fig 2) [22], which may rely on the hijacking of specific components of the membrane fusion machinery. In support of this model, targeting the ERGIC (ER-Golgi intermediate compartment) Q-SNARE Stx5 or the ER R-SNARE Sec22b and its cognate Q-SNARE partners Stx18 and D12 restricts the expansion of *L*. *amazonensis* PVs and is detrimental to parasite replication [24]. Whether the molecular basis for this fundamental difference in the lifestyle of these two groups of *Leishmania* is related to differential expression or activity of virulence factors such as GP63 that directly target specific components of the host cell machinery remains to be investigated.

## Conclusion

As our understanding of the function of membrane fusion mediators deepens, we are able to get a better insight into the challenges faced by *Leishmania* parasites upon entry into host cells and, in parallel, the mechanisms of parasite virulence and pathogenesis. Conversely, *Leishmania* represents a superb tool for the identification of novel roles for the membrane fusion machinery in macrophages, by investigating the functional consequences of host protein cleavage or intracellular redistribution on cell and immune functions. Components of the membrane fusion machinery might emerge as targets for novel therapeutic interventions in infectious and inflammatory diseases.
